# Machine learning-based time-to-event survival analysis in pediatric patients with severe sepsis

**DOI:** 10.3389/fped.2025.1688416

**Published:** 2025-10-23

**Authors:** Qianru Huang, Li Zheng, Ruyi Cai, Haiyang Chen

**Affiliations:** ^1^ The Affiliated Jiangning Hospital of Nanjing Medical University, Nanjing, China; ^2^ Lianshui People's Hospital of Kangda College Affiliated to Nanjing Medical University, Huaian, China; ^3^ Women's Hospital of Nanjing Medical University, Nanjing, China; ^4^ Huai'an TCM Hospital Affiliated to Nanjing University of Chinese Medicine, Huaian, China

**Keywords:** survival analysis, machine learning, pediatric sepsis, time-to-event, SHapley Additive exPlanations

## Abstract

**Background:**

Pediatric sepsis remains a leading cause of mortality in critically ill children worldwide. Current approaches to sepsis prognosis rely on clinical criteria and biomarkers with variable performance. This study aimed to develop and validate time-to-event survival prediction models for pediatric sepsis using survival analysis machine learning algorithms.

**Methods:**

We conducted a retrospective cohort study of 223 pediatric sepsis patients from a pediatric intensive care database (2010–2018). Five survival analysis machine learning algorithms were evaluated: CoxPHSurvivalAnalysis, HingeLossSurvivalSVM, GradientBoostingSurvivalAnalysis, RandomSurvivalForest, and ExtraSurvivalTrees. These algorithms predict survival time rather than binary outcomes. Model performance was assessed using time-dependent area under the curve (td-AUC), concordance index (c-index), Brier score, and calibration curves. SHapley Additive exPlanations (SHAP) analysis was performed for model interpretability, and zero-crossing point analysis identified clinically actionable thresholds.

**Results:**

Among 223 patients, 200 (89.7%) survived with median ICU stay of 12.2 days for survivors vs. 2.3 days for non-survivors. RandomSurvivalForest achieved the highest performance with td-AUC of 0.97, while CoxPHSurvival and HingeLossSurvivalSVM showed comparable c-indices of 0.87. SHAP analysis identified calcium total and RDW as the strongest mortality predictors. Zero-crossing point analysis established clinical thresholds: calcium total <1.10 mmol/L, RDW >15.07%, sodium <131.68 mmol/L, and pH <7.32 were associated with increased mortality risk, with U-shaped relationships observed for creatinine and lymphocytes.

**Conclusions:**

RandomSurvivalForest demonstrated superior time-to-event prediction performance for pediatric sepsis. The survival analysis approach provides dynamic risk assessment and precise timing for clinical interventions. A web-based prediction calculator was developed to facilitate clinical implementation.

## Introduction

Sepsis is one of the leading causes of morbidity and mortality in critically ill children worldwide (1), killing approximately 4,500 children annually in the United States (2) and causing 25% mortality globally among those with severe sepsis (3). Pediatric intensive care units experience particularly high rates of sepsis due to the complex medical conditions and invasive procedures that characterize critical care (4, 5). Children with severe sepsis present unique challenges for clinicians attempting to predict outcomes and stratify risk, especially when underlying conditions complicate diagnosis and treatment (6–8). Current approaches to sepsis diagnosis and prognosis rely on clinical criteria and biomarkers like procalcitonin and C-reactive protein, which demonstrate variable performance and limited predictive accuracy (9–11). The complex pathophysiology of sepsis, particularly in children with cardiac disease, makes it difficult to identify high-risk patients and predict outcomes accurately (12, 13). Better survival prediction tools are needed to help clinicians counsel families and make treatment decisions.

Machine learning (ML) have emerged as promising tools for improving sepsis outcome prediction and clinical decision-making (14–16). Recent studies have demonstrated the potential of various ML algorithms in predicting mortality among pediatric sepsis patients (17–19). Moore et al. evaluated multiple ML models including random forest, light gradient boosting machine, and Extreme Gradient Boosting for predicting in-hospital mortality in children with Phoenix sepsis, achieving area under the receiver operating characteristic curves (AUROCs) ranging from 0.81 to 0.88, with random forest showing the best performance (17). Kim et al. developed the Pediatric Risk of Mortality Prediction Tool (PROMPT) using a convolutional neural network, which achieved AUROCs of 0.89–0.97 for mortality prediction 6–60 h prior to death in critically ill children, outperforming conventional severity scoring systems (18). Additionally, Shimabukuro et al. conducted a randomized controlled trial of a ML -based severe sepsis prediction algorithm, demonstrating significant reductions in average length of stay (from 13.0 to 10.3 days, *p* = 0.042) and in-hospital mortality (12.4 percentage point reduction, *p* = 0.018) (19). These findings highlight the clinical utility of ML in sepsis management. However, very few studies have specifically focused on using ML approaches to predict survival time and survival status in children with severe sepsis.

In this study, we utilized data from a pediatric intensive care unit database to develop survival prediction models for children with severe sepsis. We compared several survival analysis machine learning algorithms using time-dependent area under the curve (td-AUC), concordance index (c-index), Brier score, and calibration curves to identify the optimal model. The best-performing model was interpreted using SHapley Additive exPlanations (SHAP) analysis, and a web-based calculator was developed for clinical application.

## Methods

### Study population

We conducted a retrospective cohort study using data from the Paediatric Intensive Care (PIC) database, including all pediatric patients diagnosed with sepsis who were admitted to intensive care units at the Children's Hospital, Zhejiang University School of Medicine between 2010 and 2018. Data access was obtained following completion of the required CITI training program (certification 64180628) and execution of the data use agreement. Patients were included if they were ≤18 years at ICU admission, had a primary or secondary diagnosis of sepsis according to International Pediatric Sepsis Consensus Conference criteria, and had complete data for survival time and vital status. We excluded patients with missing essential clinical data, ICU stay <24 h, or incomplete admission data due to transfer from other hospitals. The primary outcome was survival time from ICU admission to death or hospital discharge.

### Data extraction and preprocessing

**Figure 1:**
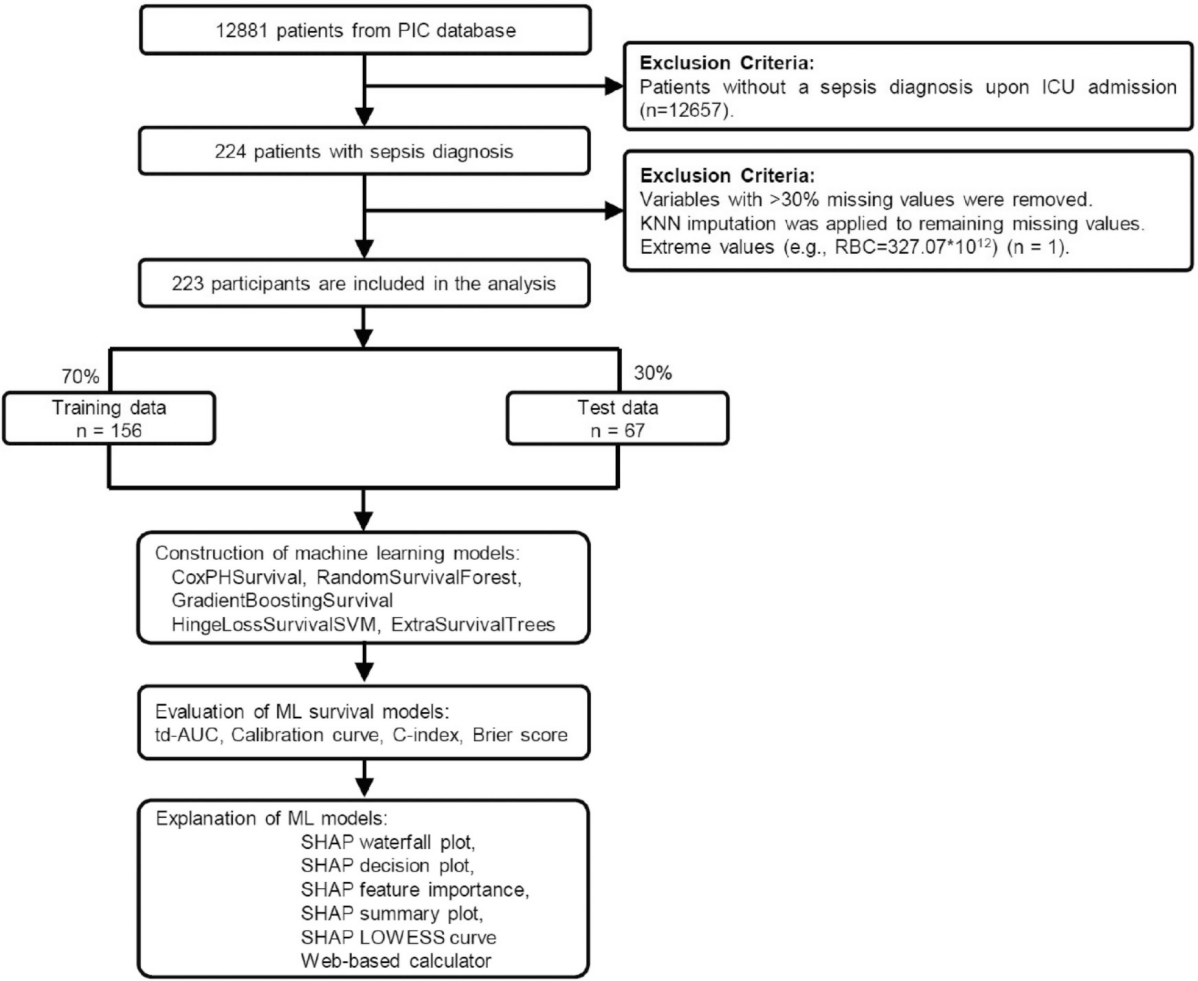
Study flowchart and machine learning pipeline for pediatric sepsis survival prediction. Patient selection from 12,881 PIC database records to 223 pediatric sepsis patients, followed by 7:3 training-testing split. Five survival analysis algorithms were evaluated using td-AUC, c-index, Brier score, and calibration curves. The optimal model underwent SHAP analysis and web-based calculator development. PIC, Paediatric Intensive Care; td-AUC, time-dependent area under the curve; SHAP, SHapley Additive exPlanations.

The study cohort was derived from 12,881 patients in the PIC database. After excluding 12,657 patients without a sepsis diagnosis upon ICU admission, 224 patients with sepsis were identified. One patient was further excluded due to extreme laboratory values (RBC = 327.07 × 10^12^/L), resulting in a final cohort of 223 participants (Figure 1). Clinical data were initially extracted from the first measurements obtained within 24 h of ICU admission, including demographic characteristics, laboratory measurements, vital signs, anthropometric measurements, medication usage, and fluid balance data. Variables with >30% missing values were removed, and K-nearest neighbors (KNN) imputation was applied to handle remaining missing values. The final dataset included age, gender, hematological parameters (RBC, WBC, neutrophil percentage, lymphocyte percentage, platelet count, hemoglobin, RDW, hematocrit), biochemical markers (sodium, potassium, calcium, chloride, ALT, creatinine), blood gas analysis (pH, PCO_2_, PO_2_, lactate), medication usage (cephalosporins, vasopressors), fluid balance data (liquid input and output), and ICU length of stay. Survival time was calculated from ICU admission to death or hospital discharge.

Continuous variables were standardized using “StandardScaler” package in Python. Variance inflation factor (VIF) was calculated to detect multicollinearity, and variables with VIF >10 were excluded (Supplementary Table S1).

Variable selection was performed using a multi-stage approach. First, univariate Cox regression analysis was conducted for all candidate variables to assess individual associations with mortality. Variables with high correlation (*r* > 0.6) were identified, and the less statistically significant variable from each correlated pair was removed to reduce multicollinearity. Final variable selection prioritized statistically significant predictors (*p* < 0.05) from univariate analysis, supplemented by variables with the strongest effect sizes based on hazard ratios. The selected variables were then incorporated into a multivariable Cox proportional hazards model, with performance assessed using Harrell's concordance index. Following this selection process, 15 variables were ultimately included in the final model: Age, Gender, RDW, Lymphocytes, Hemoglobin, Lactate, pH, PO2, Sodium, CalciumTotal, Chloride, Creatinine, Cephalosporins, Vasopressors, and Liquid input. All variables underwent normality testing for survivors, non-survivors, and the total cohort, with complete results presented in Supplementary Table S4.

### ML model construction and evaluation

The dataset was randomly split into training and testing sets using a 7:3 ratio, with 156 patients allocated to the training set and 67 patients to the testing set. Stratified sampling was employed to ensure balanced distribution of survival outcomes between the two sets. Five survival analysis machine learning algorithms were implemented and evaluated for survival prediction, including CoxPHSurvivalAnalysis (a regularized Cox proportional hazards model), HingeLossSurvivalSVM (Support Vector Machine adapted for survival analysis using hinge loss function), GradientBoostingSurvivalAnalysis (gradient boosting algorithm for survival data), RandomSurvivalForest (ensemble method extending random forests to survival analysis), and ExtraSurvivalTrees (extremely randomized survival trees with additional randomness in threshold selection). For each algorithm, comprehensive hyperparameter optimization was performed using 10-fold cross-validation with grid search on the training set. The hyperparameter search spaces included alpha, n_iter, ties, and tol for CoxPHSurvivalAnalysis; alpha, solver types, kernel functions, constraint pairs, and maximum iterations for HingeLossSurvivalSVM; number of estimators, maximum depth, minimum samples for splitting and leaf nodes for the tree-based ensemble methods including GradientBoostingSurvivalAnalysis, RandomSurvivalForest, and ExtraSurvivalTrees, with ExtraSurvivalTrees additionally optimizing maximum leaf nodes parameters (Supplementary Table S2).

Model performance was evaluated using time-dependent Area Under the Curve (td-AUC) calculated at multiple time points to assess discriminative ability over time, concordance index (C-index) to measure the probability that predicted survival rankings align with observed survival times, Brier score to evaluate prediction accuracy as a proper scoring rule for survival models, and calibration curves to assess agreement between predicted and observed survival probabilities. All models were implemented using the scikit-survival library in Python 3.12, with optimal hyperparameters selected based on the highest C-index achieved during cross-validation on the training set, and final model evaluation performed on the independent testing set to assess generalization performance.

### SHAP analysis

To enhance model interpretability and understand feature contributions, SHapley Additive exPlanations (SHAP) analysis was performed on the best-performing model. A KernelExplainer was initialized using K-means clustering with 50 cluster centers as background data to reduce computational complexity while maintaining representative coverage of the feature space. Feature importance was determined by calculating the mean absolute SHAP values across all samples, and the top 8 most influential features were identified for detailed analysis. Model interpretability was visualized through multiple SHAP plots including waterfall plots to show individual prediction explanations, summary plots to display feature importance rankings, and partial dependence plots with LOWESS (Locally Weighted Scatterplot Smoothing) regression to illustrate the relationship between feature values and SHAP contributions. Zero-crossing points were identified and marked to determine optimal thresholds where features transition from protective to harmful effects on survival outcomes.

### Web-based calculator

For clinical applications, a web-based survival prediction calculator was developed using the Gradio framework. The interface accepts all model input variables including demographic data, laboratory parameters, medication usage, and fluid balance information. The trained model and preprocessing scaler were integrated to provide real-time survival predictions with probability curves for the first 7 days of ICU stay and survival probabilities at key time points (1, 3, 5, and 7 days), making the predictive model accessible for clinical decision support (https://huggingface.co/spaces/MLlab00/sepsis). The tool processes data locally without storing or recording any patient information, ensuring privacy protection.

### Statistical analysis

Continuous variables were assessed for normality using the Shapiro–Wilk test within each group. Normally distributed continuous variables were presented as mean ± standard deviation (SD), while non-normally distributed continuous variables were expressed as median (25th percentile, 75th percentile). Categorical variables were expressed as frequency and percentage (*n*, %). For group comparisons, independent *t*-tests were used for normally distributed continuous data and Mann–Whitney *U* tests for non-normally distributed continuous data. Categorical variables were compared using Fisher's exact test or chi-square tests as appropriate. Statistical significance was set at *p* < 0.05. All statistical analyses were performed using R version 4.4 and Python version 3.12.

## Results

### Baseline characteristics

A total of 223 pediatric patients with sepsis were included in the final analysis, with 200 (89.7%) survivors and 23 (10.3%) non-survivors. The baseline characteristics are presented in Table 1. The median age was 0.1 (0.0, 0.2) years with no significant difference between groups (*p* = 0.145), and gender distribution was similar (60.5% male, *p* = 1.0). Most hematological and biochemical parameters showed no significant differences between survivors and non-survivors, except for red cell distribution width (15.9 vs. 14.9, *p* = 0.004), total calcium levels (1.2 vs. 1.1 mmol/L, *p* = 0.02), chloride levels (109.0 vs. 105.2 mmol/L, *p* = 0.012), and pH values (7.4 vs. 7.3, *p* = 0.005).

**Table 1:** Baseline characteristics of patients.

Variable	Survivors	Non-survivors	Total	*p* value
	(*N* = 200)	(*N* = 23)	(*N* = 223)	
Age, years	0.1 (0.0, 0.2)	0.1 (0.1, 0.4)	0.1 (0.0, 0.2)	0.145
Gender, *n* (%)				1
Male	121 (60.5%)	14 (60.9%)	135 (60.5%)	
Female	79 (39.5%)	9 (39.1%)	88 (39.5%)	
RDW	15.9 (15.1, 17.2)	14.9 (13.7, 15.7)	15.8 (14.8, 17.1)	0.004
Lymphocytes, %	27.2 (18.8, 38.2)	27.2 (21.5, 48.0)	27.2 (19.1, 39.8)	0.286
Hemoglobin, g/L	116.2 (93.9, 135.3)	104.0 (90.2, 115.8)	113.5 (93.5, 135.0)	0.16
Lactate, mmol/L	2.1 (1.4, 3.5)	5.2 (2.4, 6.1)	2.2 (1.5, 3.7)	<0.001
pH	7.4 (7.3, 7.4)	7.3 (7.2, 7.4)	7.4 (7.3, 7.4)	0.005
PO2, mmHg	102.7 (80.2, 125.0)	102.3 (74.6, 128.0)	102.5 (79.9, 125.0)	0.852
Sodium, mmol/L	136.0 (133.5, 139.0)	135.0 (130.2, 137.2)	136.0 (133.0, 139.0)	0.137
Calcium, mmol/L	1.2 (1.1, 1.2)	1.1 (1.0, 1.2)	1.2 (1.1, 1.2)	0.02
Chloride, mmol/L	109.0 (105.5, 112.5)	105.2 (102.7, 109.1)	108.0 (105.2, 112.4)	0.012
Creatinine, μmol/L	55.5 (40.0, 79.2)	40.4 (31.0, 73.0)	55.0 (39.0, 79.0)	0.15
Cephalosporins, *n* (%)	76 (38.0%)	3 (13.0%)	79 (35.4%)	0.02
Vasopressors, *n* (%)	62 (31.0%)	13 (56.5%)	75 (33.6%)	0.026
Liquid input, ml	0.0 (0.0, 184.5)	0.0 (0.0, 0.0)	0.0 (0.0, 166.5)	<0.001
ICU stay, days	12.2 (4.9, 25.7)	2.3 (1.6, 3.3)	11.0 (3.6, 21.9)	<0.001

Notable differences were observed in lactate levels, which were significantly higher in non-survivors (5.2 vs. 2.1 mmol/L, *p* < 0.001). Cephalosporin usage was more frequent in survivors (38.0% vs. 13.0%, *p* = 0.02), while vasopressor usage was more frequent in non-survivors (56.5% vs. 31.0%, *p* = 0.026). Survivors had significantly more liquid input (0.0 vs. 0.0 ml, *p* < 0.001) and longer ICU stays (12.2 vs. 2.3 days, *p* < 0.001).

### Survival machine learning model performance evaluation

**Figure 2:**
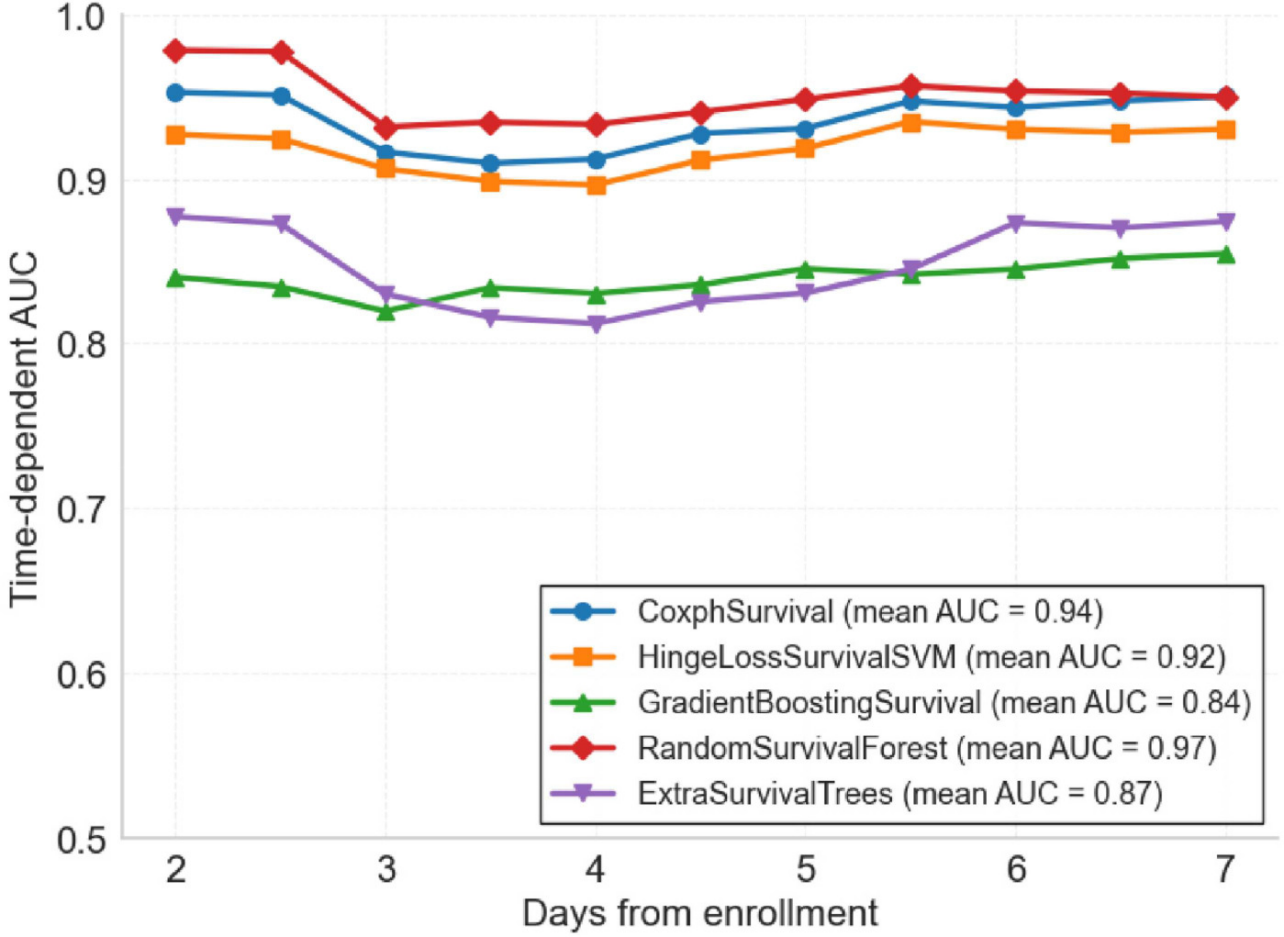
Time-dependent area under the curve (td-AUC) comparison of five survival analysis machine learning algorithms. Performance of each model from day 2 to day 7 after ICU enrollment. RandomSurvivalForest achieved the highest mean AUC of 0.97, followed by CoxphSurvival (0.94), HingeLossSurvivalSVM (0.92), ExtraSurvivalTrees (0.87), and GradientBoostingSurvival (0.84).

Five survival analysis machine learning algorithms were evaluated and compared for their predictive performance. Time-dependent AUC analysis revealed significant differences in discriminative ability across models (Figure 2). RandomSurvivalForest demonstrated superior performance with the highest mean time-dependent AUC of 0.97, maintaining consistently high discriminative ability across all time points from day 2 to day 7. CoxphSurvival and HingeLossSurvivalSVM showed comparable performance with mean AUCs of 0.94 and 0.92 respectively, both maintaining stable predictive accuracy throughout the observation period. ExtraSurvivalTrees achieved a mean AUC of 0.87, while GradientBoostingSurvival exhibited the lowest performance with a mean AUC of 0.84, showing consistently lower discriminative ability compared to the other models. Detailed time-dependent AUC values for each day are presented in Supplementary Table S3.

**Figure 3:**
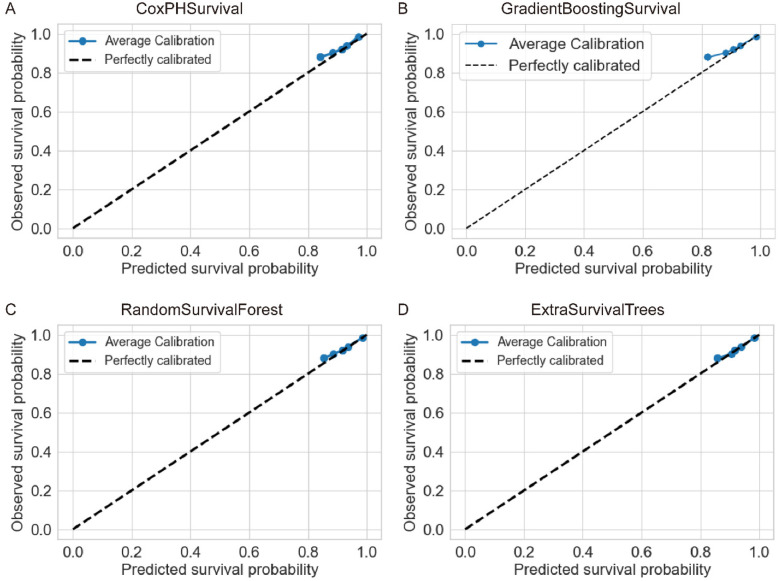
Calibration curves for four survival analysis machine learning algorithms. Each panel shows the agreement between predicted and observed survival probabilities for **(A)** CoxPHSurvival, **(B)** GradientBoostingSurvival, **(C)** RandomSurvivalForest, and **(D)** ExtraSurvivalTrees. The dashed diagonal line represents perfect calibration. ExtraSurvivalTrees demonstrated the best calibration performance with points closely following the ideal diagonal line.

Further evaluation using concordance indices and Brier scores confirmed the superior performance of RandomSurvivalForest (Table 2). CoxPHSurvival and HingeLossSurvivalSVM achieved the highest c-index of 0.87 (95% CI: 0.77–0.95 and 0.76–0.95, respectively), while GradientBoostingSurvival, RandomSurvivalForest, and ExtraSurvivalTrees showed comparable performance with c-indices of 0.85 (95% CI: 0.64–0.95, 0.65–0.96, and 0.65–0.96, respectively). Brier scores were consistently low across the evaluated models (ranging from 0.07 to 0.08), indicating good overall prediction accuracy. Note that Brier score was not available for HingeLossSurvivalSVM. Calibration analysis showed varying performance across models (Figure 3), with ExtraSurvivalTrees and RandomSurvivalForest demonstrating excellent agreement between predicted and observed survival probabilities, closely following the ideal diagonal line, while CoxPHSurvival and GradientBoostingSurvival showed suboptimal calibration with greater deviations from perfect calibration. Based on the combination of highest time-dependent AUC, highest c-index, and excellent calibration, RandomSurvivalForest was selected as the optimal model for subsequent SHAP analysis and clinical application.

**Table 2:** Discriminative ability and calibration of each model.

Models	c-index (95% CI)	Brier score (95% CI)
CoxPHSurvival	0.87 (0.77–0.95)	0.07 (0.02–0.14)
HingeLossSurvivalSVM	0.87 (0.76–0.95)	/
GradientBoostingSurvival	0.85 (0.64–0.95)	0.08 (0.03–0.15)
RandomSurvivalForest	0.85 (0.65–0.96)	0.08 (0.03–0.15)
ExtraSurvivalTrees	0.85 (0.65–0.96)	0.08 (0.03–0.15)

### SHAP model interpretation

**Figure 4:**
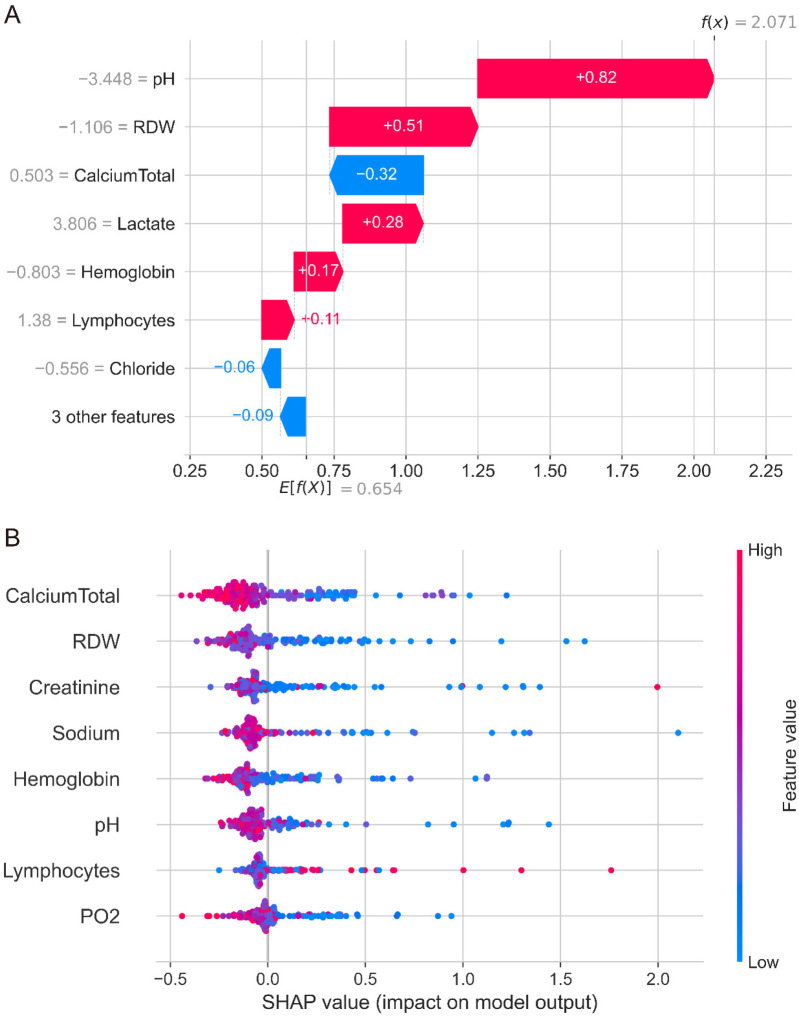
SHAP analysis of the randomSurvivalForest model. **(A)** Waterfall plot showing individual feature contributions for a representative patient case. pH provides the strongest risk contribution (SHAP value +0.82), while calcium total provides a protective effect (SHAP value −0.32). Other notable contributors include RDW (+0.51), lactate (+0.28), and hemoglobin (+0.17). **(B)** Summary plot displaying feature importance and value distributions across all patients. Each point represents one patient, with colors indicating high (red) to low (blue) feature values. Calcium total and RDW emerged as the most influential predictors, with complex patterns showing variable contributions across different patient populations.

SHAP analysis was performed on the optimal RandomSurvivalForest model to enhance interpretability and identify key predictive features. Feature importance analysis revealed that calcium total and RDW were the two most influential variables, followed by creatinine, sodium, and hemoglobin. Other important predictors included pH, lymphocytes, PO2, chloride, and lactate (Supplementary Figure S1A). The waterfall plot for a representative case demonstrated how individual features contributed to the final prediction, with pH providing the strongest risk contribution (SHAP value: +0.82) and calcium total providing a protective effect (SHAP value: −0.32) for this specific patient (Figure 4A). Other notable contributors included RDW (+0.51), lactate (+0.28), and hemoglobin (+0.17), illustrating how multiple clinical parameters collectively influenced this specific patient (Figure 4A).

The SHAP summary plot revealed complex patterns in feature contributions across the patient cohort (Figure 4B). While calcium total levels generally showed protective effects with negative SHAP values, there was considerable variability across patients. Similarly, RDW, creatinine, sodium, and other continuous variables demonstrated overlapping distributions of positive and negative SHAP values, indicating that the relationship between these features and mortality risk varies significantly across different patients and clinical contexts. This complexity suggests that the interactions between variables and non-linear relationships may play crucial roles in mortality prediction, warranting further detailed analysis of each variable's specific contribution patterns and threshold effects in the model. The decision plot visualization (Supplementary Figure S1B) further illustrated the cumulative effect of all features on model predictions, showing how different combinations of clinical variables led to varying survival predictions across the patient population.

**Figure 5:**
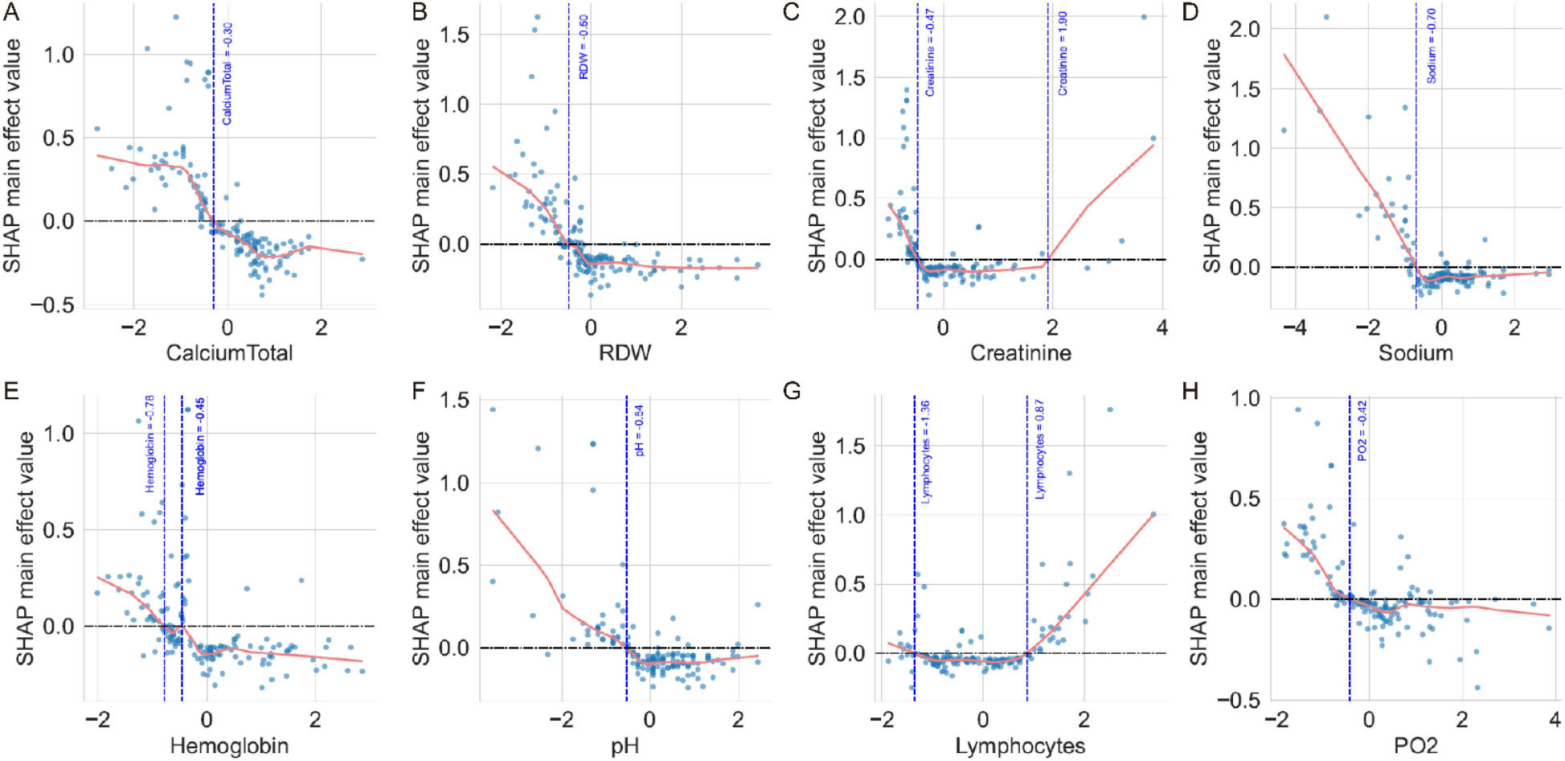
SHAP feature value plots with zero-crossing point analysis. **(A)** Calcium total showing a zero-crossing point at 1.10 mmol/L, where values below this threshold contribute to increased mortality risk. **(B)** RDW demonstrates a zero-crossing point at 15.07%, with higher values associated with increased mortality risk. **(C)** Creatinine shows zero-crossing points at 42.0 μmol/L and 170.33 μmol/L, indicating a U-shaped relationship with mortality risk. **(D)** Sodium exhibits a zero-crossing point at 131.68 mmol/L, with lower values contributing to increased mortality risk. **(E)** Hemoglobin demonstrates zero-crossing points at 102.65 g/L and 91.75 g/L, showing complex threshold effects. **(F)** pH shows a zero-crossing point at 7.32, with acidosis contributing to increased mortality risk. **(G)** Lymphocytes exhibit zero-crossing points at 10.11% and 43.39%, indicating optimal ranges for survival. **(H)** PO2 shows a zero-crossing point at 89.08 mmHg, with lower values associated with increased mortality risk. The horizontal dashed line represents SHAP value = 0, and vertical dashed lines mark the zero-crossing thresholds identified by LOWESS regression curves.

SHAP feature value plots with LOWESS regression identified critical thresholds where SHAP contributions transition from negative to positive values (Figure 5). For continuous variables, zero-crossing points revealed clinically relevant cutoff values: calcium total at 1.10 mmol/L (Figure 5A), RDW at 15.07% (Figure 5B), creatinine showing dual thresholds at 42.0 μmol/L and 170.33 μmol/L (Figure 5C), sodium at 131.68 mmol/L (Figure 5D), hemoglobin with dual thresholds at 91.75 g/L and 102.65 g/L (Figure 5E), pH at 7.32 (Figure 5F), lymphocytes with dual thresholds at 10.11% and 43.39% (Figure 5G), and PO2 at 89.08 mmHg (Figure 5H). These thresholds demonstrate that values below the cutoffs for calcium total, sodium, hemoglobin, pH, and PO2 contribute to increased mortality risk, while RDW values above 15.07% are associated with higher mortality risk. The dual thresholds observed for creatinine and lymphocytes suggest U-shaped relationships, indicating optimal physiological ranges for survival outcomes in pediatric sepsis management.

### Web-based survival prediction calculator

To facilitate clinical implementation, a user-friendly web-based survival prediction calculator was developed and deployed using the optimal RandomSurvivalForest model (Supplementary Figure S2). The calculator interface allows clinicians to input patient-specific clinical parameters including demographic information, laboratory values, medication usage, and fluid balance data. Upon entering the required variables, the system automatically generates personalized survival predictions with probability estimates at key time points (Day 1: 100.0%, Day 3: 74.0%, Day 5: 74.0%, Day 7: 74.0% in the demonstrated case) and displays a comprehensive survival probability curve for the first 7 days of ICU stay. The calculator provides immediate risk assessment and supports clinical decision-making by offering quantitative survival estimates that can inform treatment planning and family counseling in pediatric sepsis management.

## Discussion

In this retrospective cohort study of 223 pediatric sepsis patients, we found that RandomSurvivalForest achieved the best predictive performance among five survival analysis algorithms, with a time-dependent AUC of 0.97 and superior calibration compared to other models. Our SHAP analysis identified calcium total and RDW as the strongest predictors of mortality risk, with complex threshold effects revealed through zero-crossing point analysis. Specifically, calcium total levels below 1.10 mmol/L, RDW values above 15.07%, sodium levels below 131.68 mmol/L, and pH values below 7.32 were associated with significantly increased mortality risk. The analysis also revealed U-shaped relationships for creatinine (thresholds at 42.0 and 170.33 μmol/L) and lymphocytes (thresholds at 10.11% and 43.39%), indicating optimal physiological ranges for survival outcomes. The web-based prediction calculator we developed provides clinicians with immediate access to personalized survival probabilities, potentially improving risk assessment and treatment planning in pediatric intensive care settings.

Our study demonstrates superior predictive performance compared to previous machine learning applications in pediatric sepsis mortality prediction. While most existing studies have focused on predicting mortality at fixed time points, our approach represents the first application of survival analysis algorithms in this population, providing dynamic risk assessment over time rather than static predictions. Banerjee et al. achieved an AUC of 0.82 using gene expression profiles from 228 septic patients in PICU settings, while their external validation showed variable performance (AUC: 0.72–0.96 across different datasets) (20). Zhou et al. developed a CatBoost model for sepsis-associated acute kidney injury patients with an AUC of 0.83 (21), and Hsu et al. reported an AUC of 0.923 using deep neural networks in neonatal sepsis (22). In contrast, our RandomSurvivalForest model achieved a consistently higher time-dependent AUC of 0.97 with excellent calibration across all time points. This approach offers more precise timing for clinical interventions compared to binary outcome predictions.

This study represents the first application of survival analysis machine learning algorithms in pediatric sepsis, providing time-to-event predictions rather than traditional binary outcomes. We implemented SHAP interpretability analysis to explain individual predictions and identify feature contributions, while zero-crossing point analysis using LOWESS regression established clinically actionable thresholds for biomarkers. Our approach included rigorous hyperparameter optimization with 10-fold cross-validation and comprehensive evaluation across multiple performance metrics. The development of an immediately deployable web-based prediction tool bridges the gap between research and clinical practice, providing frontline clinicians with accessible predictive analytics for critically ill children.

The limited number of non-survivors (*n* = 23, 10.3%) significantly constrains the precision of our model's performance estimates. This is exemplified by the wide confidence interval of our C-index (0.87, 95% CI: 0.76–0.95), indicating substantial uncertainty in the model's discriminatory ability, with true performance potentially ranging from barely better than random prediction to excellent discrimination. Additionally, with an event-per-variable ratio of 7.7 (below the recommended 10–15 threshold), our model is susceptible to overfitting, which may explain the high td-AUC of 0.96. The small sample size also limits our statistical power to detect clinically meaningful risk gradations, with a minimum detectable difference of approximately 8%–10% in predicted mortality probability. These limitations collectively suggest that larger, more balanced cohorts are needed to establish reliable performance estimates and clinical utility.

Our established thresholds for calcium levels provide actionable clinical guidance. The calcium threshold of 1.1 mmol/L suggests that hypocalcemia correction should be prioritized in pediatric sepsis management. Calcium homeostasis is essential for maintaining normal myocardial contraction/relaxation cycles, and hypocalcemia has been associated with cardiovascular dysfunction, acute kidney injury, coagulopathy, and increased mortality in septic patients (23–25). Our findings are consistent with previous studies in pediatric populations, where Zheng et al. demonstrated that ionized calcium was an independent predictor of poor prognosis in very low birth weight infants with sepsis (OR: 0.283, 95% CI: 0.126–0.638, *p* = 0.002) (23), further supporting the critical role of calcium homeostasis in pediatric sepsis outcomes.

The observational nature of our data precludes causal inference due to confounding by indication, where treatment decisions are influenced by patient severity and clinical judgment (26, 27). To establish causal relationships, future research should employ causal inference methodologies such as target trial emulation, instrumental variable analysis, or propensity score approaches that can better isolate treatment effects from confounding factors. Only through rigorous causal analysis can we determine whether interventions targeting these modifiable factors can improve outcomes, transforming predictive associations into actionable clinical guidance for pediatric sepsis management (28).

An important consideration in interpreting our findings is the inherent heterogeneity of pediatric sepsis. Sepsis represents a complex syndrome with diverse underlying etiologies, varying host responses, and distinct clinical phenotypes that may exhibit different prognostic patterns (29, 30). Our study population encompassed patients with varied infection sources, age ranges, and degrees of organ dysfunction, yet we applied a unified predictive model without stratification by clinical subgroups or sepsis endotypes. The clinical thresholds identified through SHAP analysis may not apply uniformly across all sepsis phenotypes, as neonatal sepsis may demonstrate different physiological responses compared to older pediatric patients, and gram-positive vs. gram-negative infections may present distinct biomarker patterns (31). Future research should investigate model performance across specific sepsis subgroups to enable development of more personalized risk assessment tools and advance precision medicine in pediatric sepsis care.

Several limitations should be acknowledged in this study. First, this was a single-center retrospective study, which may limit the generalizability of our findings to other pediatric populations and healthcare settings. The relatively small sample size of 223 patients, particularly the low number of non-survivors (*n* = 23), may affect model stability and the precision of risk estimates. The study period spanning 2010–2018 may introduce temporal bias due to changes in clinical practice and treatment protocols over time. Additionally, we excluded patients with ICU stays <24 h, which may have removed some early deaths and introduced selection bias. The dataset lacked important clinical variables such as pediatric-specific severity scores (PRISM, PELOD, pSOFA), source of infection, antibiotic resistance patterns, and detailed organ support measures, which could improve model performance. External validation in independent cohorts is needed to confirm the robustness and generalizability of our findings before widespread clinical implementation.

## Conclusion

This study developed a survival prediction model for pediatric sepsis using RandomSurvivalForest, achieving a time-dependent AUC of 0.97. SHAP analysis identified calcium total and RDW as the strongest mortality predictors, while establishing clinically relevant thresholds including calcium total <1.10 mmol/L, RDW >15.07%, sodium <131.68 mmol/L, and pH <7.32 as indicators of increased mortality risk. Additional U-shaped relationships were identified for creatinine and lymphocytes, revealing optimal physiological ranges for survival outcomes. The web-based prediction calculator provides clinicians with immediate access to survival probability estimates for risk stratification and clinical decision-making in pediatric sepsis management.

## Data Availability

The raw data supporting the conclusions of this article will be made available by the authors, without undue reservation.

## Supplementary material

Supplementary Figure S1 SHAP feature importance and decision plot analysis. (A) Bar plot showing the top 10 features ranked by mean absolute SHAP values. Calcium total and RDW are the most influential predictors, followed by creatinine, sodium, hemoglobin, pH, lymphocytes, PO2, chloride, and lactate. (B) Decision plot illustrating the cumulative effect of all features on model predictions for individual patients. Each line represents one patient's prediction path from the expected value (left) to the final model output (right), showing how different feature combinations lead to varying survival predictions.

Supplementary Figure S2 Web-based survival prediction calculator interface. Screenshot of the deployed calculator showing the input interface for clinical parameters and the prediction output. The example case displays survival probabilities at key time points (Day 1: 100.0%, Day 3: 74.0%, Day 5: 74.0%, Day 7: 74.0%) and a survival probability curve for the first 7 days of ICU stay. The calculator accepts demographic data, laboratory values, medication usage, and fluid balance information to generate personalized survival predictions.
