# Supplementary figures and images for: Machine learning-based time-to-event survival analysis in pediatric patients with severe sepsis
Source: Front Pediatr. 2025 Oct 23;13:1688416. doi: 10.3389/fped.2025.1688416 (PMC12589007; doi:10.3389/fped.2025.1688416)

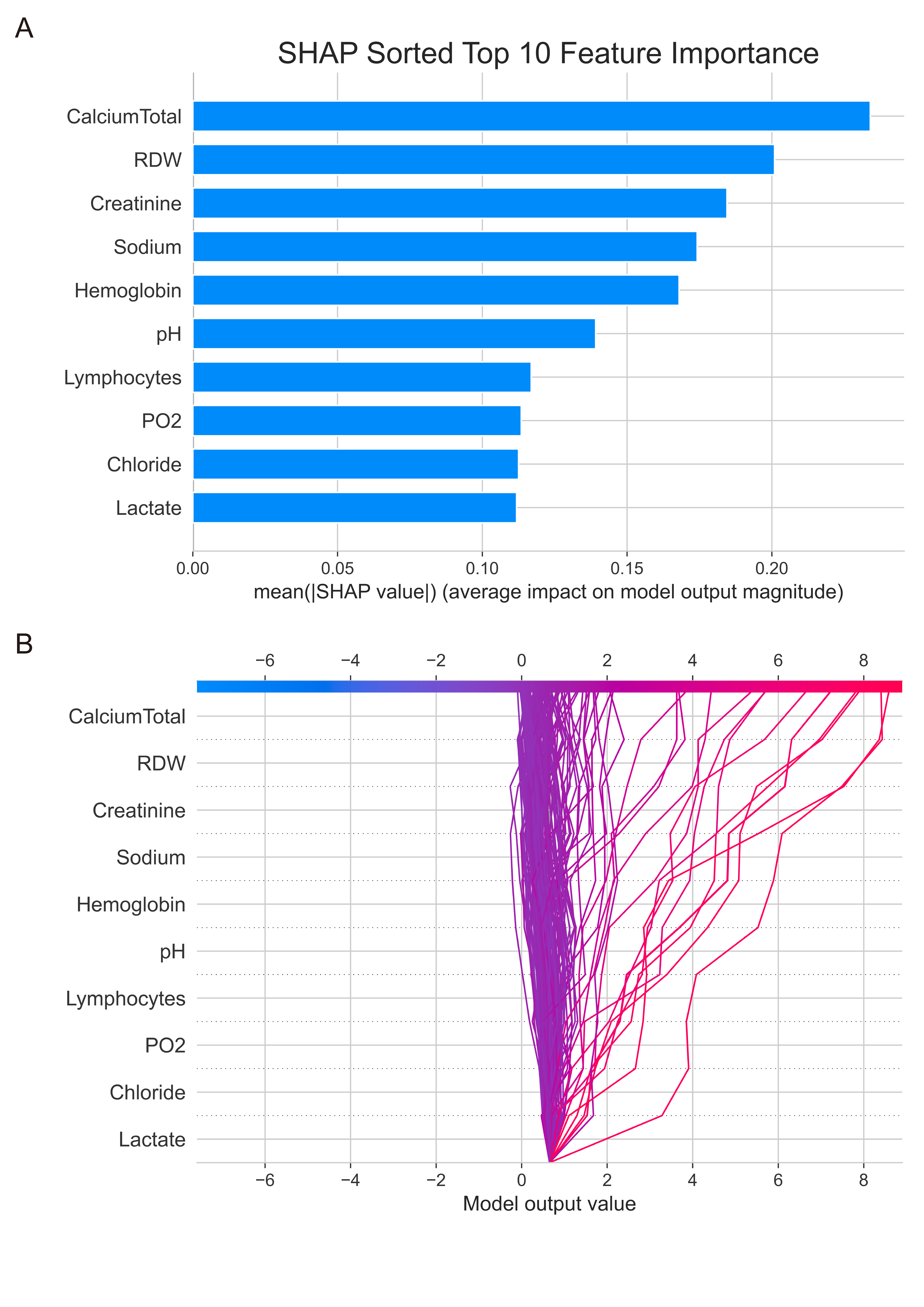

Supplement: Supplementary Figure S1 — SHAP feature importance and decision plot analysis. (A) Bar plot showing the top 10 features ranked by mean absolute SHAP values. Calcium total and RDW are the most influential predictors, followed by creatinine, sodium, hemoglobin, pH, lymphocytes, PO2, chloride, and lactate. (B) Decision plot illustrating the cumulative effect of all features on model predictions for individual patients. Each line represents one patient's prediction path from the expected value (left) to the final model output (right), showing how different feature combinations lead to varying survival predictions. [file Image1.tif]
